# The dynamic relationship between sleep and psychotic experiences across the early stages of the psychosis continuum

**DOI:** 10.1017/S0033291723001459

**Published:** 2023-12

**Authors:** S. van der Tuin, S. H. Booij, A. J. Oldehinkel, D. van den Berg, J. T. W. Wigman, U. Lång, I. Kelleher

**Affiliations:** 1 University of Groningen, University Medical Center Groningen, Department of Psychiatry, Interdisciplinary Centre Psychopathology and Emotion Regulation, Groningen, the Netherlands; 2 Center for Integrative Psychiatry, Lentis, Groningen, the Netherlands; 3 Department of Clinical, Neuro- and Developmental Psychology, Amsterdam Public Health Research Institute, Vrije Universiteit Amsterdam, Amsterdam, Netherlands; 4 Department of Psychosis Research, Parnassia Psychiatric Institute, The Hague, the Netherlands; 5 University College Dublin, School of Medicine, Dublin, Ireland; 6 University of Edinburgh, Centre for Clinical Brain Sciences, Edinburgh, UK

**Keywords:** Clinical staging model, daily diary data, psychotic experiences, sleep

## Abstract

**Background:**

Psychotic disorders develop gradually along a continuum of severity. Understanding factors associated with psychosis development, such as sleep, could aid in identification of individuals at elevated risk. This study aimed to assess (1) the dynamic relationship between psychotic experiences (PEs) and sleep quality and quantity, and (2) whether this relationship differed between different clinical stages along the psychosis continuum.

**Methods:**

We used daily diary data (90 days) of individuals (*N* = 96) at early stages (i.e. before a first diagnosis of psychosis) along the psychosis continuum. Multilevel models were constructed with sleep quality and sleep quantity as predictors of PEs and vice versa. Post-hoc, we constructed a multilevel model with both sleep quality and quantity as predictors of PEs. In addition, we tested whether associations differed between clinical stages.

**Results:**

Within persons, poorer sleep predicted next day PEs (*B* = −0.02, *p* = 0.01), but not vice versa. Between persons, shorter sleep over the 90-day period predicted more PEs (*B* = −0.04, *p* = 0.002). Experiencing more PEs over 90-days predicted poorer (*B* = −0.02, *p* = 0.02) and shorter (*B* = −1.06, *p* = 0.008) sleep. We did not find any significant moderation effects for clinical stage.

**Conclusions:**

We found a bidirectional relationship between sleep and PEs with daily fluctuations in sleep predicting next day PEs and general patterns of more PEs predicting poorer and shorter sleep. Our results highlight the importance of assessing sleep as a risk marker in the early clinical stages for psychosis.

## Introduction

Psychotic disorders affect approximately 3% of the population (van Os, Linscott, Myin-Germeys, Delespaul, & Krabbendam, 2009) and are associated with substantial morbidity, as well as increased mortality (Buckley, Miller, Lehrer, & Castle, 2009; Kelleher, Ramsay, & DeVylder, 2017). A substantially larger proportion of the adult population, 5 to 8%, report subclinical features of psychosis, typically termed psychotic experiences (PEs). These PEs exist along a continuum with mild, non-distressing unusual perceptual experiences or overvalued beliefs on one end and clinical psychotic disorders on the other. The clinical staging model acknowledges this continuum by defining different stages of illness severity ranging from stage 0 (at increased risk) to stage 4 (severe and unremitting illness) (McGorry, Hickie, Yung, Pantelis, & Jackson, 2006). Intervention in early stages, i.e. before the onset of a full-blown psychotic disorder, may improve prognosis, as early treatment is often more effective and less invasive in nature (e.g. lower medication dosage; McGorry et al., 2006). Early intervention requires thorough understanding of risk factors, and benefits from identification of risk markers that characterize individuals at elevated risk. One such risk factor of interest is sleep.

Sleep problems are associated with a variety of mental disorders (Freeman, Sheaves, Waite, Harvey, & Harrison, 2020; Pandi-Perumal et al., 2020), including psychosis (Brederoo, de Boer, de Vries, Linszen, & Sommer, 2021; Waite, Sheaves, Isham, Reeve, & Freeman, 2020). Several cross-sectional studies have shown a relationship between sleep problems and PEs such as paranoia and hallucinations (e.g. Freeman et al., 2010; Goines et al., 2019; Peña-Falcón, Pascualena-Nagore, and Perona-Garcelán, 2018; Reeve, Sheaves, and Freeman, 2019). Experimental studies showed that sleep deprivation (ranging from no sleep to maximum 4 h sleep) led to more PEs in healthy volunteers (Meyhöfer, Kumari, Hill, Petrovsky, & Ettinger, 2017; Petrovsky et al., 2014; Reeve, Emsley, Sheaves, & Freeman, 2018*a*), providing evidence for a causal relationship between sleep deprivation and PEs. Longitudinal research has shown that sleep problems are associated with later PEs in UHR youth (Lunsford-Avery, LeBourgeois, Gupta, & Mittal, 2015), the general population (Freeman et al., 2012; Reeve & Bell, 2022; Wang et al., 2023; Zhou et al., 2022) and patients diagnosed with non-affective psychotic disorder (Reeve, Nickless, Sheaves, & Freeman, 2018*b*). In addition, this relationship was found to be bidirectional in patients with non-affective psychosis (Reeve et al., 2018*b*), but not in adolescents from the general population (Zhou et al., 2022). Furthermore, insomnia significantly predicted the persistence of paranoia over the course of three months in a clinical adolescents sample (Bird, Waite, Rowsell, Fergusson, & Freeman, 2017). However, there can be great fluctuations in both sleep and PEs, and thus an approach that acknowledges these dynamic fluctuations over time might be better suited to examine the relationship between sleep and PEs, including the direction of this relationship. These type of analyses require detailed longitudinal data on both sleep and PEs to assess their associations over time.

Several recent studies used experience sampling methodology to study the association between sleep and PEs in samples of patients with schizophrenia (Meyer et al., 2021; Mulligan, Haddock, Emsley, Neil, & Kyle, 2016), combined patient and healthy control samples (Kammerer, Mehl, Ludwig, & Lincoln, 2021; Kasanova, Hajdúk, Thewissen, & Myin-Germeys, 2020), and general population samples (Hennig & Lincoln, 2018; Hennig, Schlier, & Lincoln, 2020). These studies found, overall, a relationship between PEs and poor sleep. Findings on the directionality of this relationship, however, have been mixed. To our knowledge, no study to date has investigated the potentially bidirectional relationship between sleep and PEs in individuals across different early clinical stages of the psychosis continuum, and whether associations differ between stages. The level of psychopathology increases with stage and different treatments are indicated for individuals in different stages (McGorry et al., 2006). Subsequently, it is possible that different mechanisms that underlie the symptoms are at play in different stages. For example, poor sleep as a potential stressor for increased PEs might be more influential in individuals in higher clinical stages as they have an increased vulnerability. PEs is less common in the earliest stages, and thus experiencing PEs might have a more detrimental effect on sleep. Discovering how the relationship between PEs and sleep may differ across these stages can stand to benefit early interventions for individuals at increased risk of developing psychosis. Most studies to date that collected time-intensive longitudinal data had short measurement periods of 6 to 14 days and thus may be more affected by specific life events. This makes the data less representative of ‘normal daily life’ compared to when symptoms are measured over a longer period. In addition, when looking at the effect of sleep on PEs, most studies (Hennig & Lincoln, 2018; Hennig et al., 2020; Kammerer et al., 2021; Meyer et al., 2021; Mulligan et al., 2016) failed to adjust for PEs at the previous time point, which might generate spurious associations. Finally, it remains unclear whether is it sleep *quality* (how individuals experience their sleep) or sleep *duration* (how long individuals sleep) that is most predictive of PEs.

To address these issues, we aimed to investigate (1) the bidirectional relationship between PEs and sleep quality (how well did you sleep) and quantity (how many hours did you sleep) with daily diary data (90 days) in individuals at various stages on the psychosis continuum, and (2) whether the association between sleep and PEs differed for individuals at different early clinical stages. Our sample consisted of individuals in early clinical stages: stage 0 (at increased risk for psychosis but no current symptoms), early-stage 1a (mild, non-specific symptoms), late-stage 1a (moderate symptoms) and stage 1b (Ultra-High Risk; UHR for psychosis), derived from the clinical staging model (McGorry et al., 2006; McGorry, Nelson, Goldstone, & Yung, 2010). We hypothesized that fluctuations in both sleep quality and quantity would be related to PEs the following day, and that daily fluctuations in PEs would affect both sleep quality and quantity the following night. We also hypothesized that these relationships would be different for individuals in different clinical stages along the psychosis continuum. As this is a first exploration using these early clinical stages, we do not have a hypothesis on the direction of this relationship nor on how it would differ across the stages. This study was preregistered on the Open Science Framework (link: https://osf.io/f4m7e/?view_only=d9066e62ce064cd493c45ea78be64102).

## Methods

### Participants and study design

**Figure 1.**
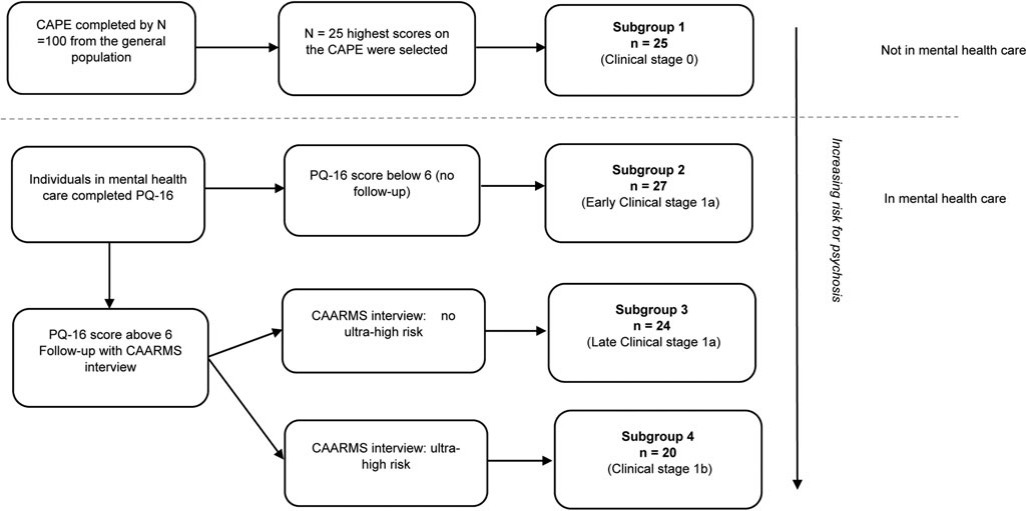
Allocation to subgroups.

Data from the Mapping Individual Routes of Risk and Resilience (Mirorr) were used (Booij et al., 2018). Mirorr combines a daily diary study with three long-term follow-up measurements on mental health and functioning in individuals at risk for psychosis. Mirorr consists of four subgroups, with each subgroup having a higher risk for psychosis; different early clinical stages for psychosis ranging from stage 0 (lowest risk) to stage 1b (Ultra-High Risk (UHR) for psychosis) (Fig. 1). Inclusion criteria were: age between 18 and 35 years, ability to read and speak Dutch fluently and to follow the research procedures, and providing informed consent. Exclusion criteria were: a history of/or current psychotic episode according to the Diagnostic and Statistical manual of Mental Disorders 4 (DSM-4) criteria, significant hearing or visual problem impairments, and pregnancy. Patients were excluded from the Mirorr study and replaced when they had more than 21 missing data points, or more than 5 subsequent missing data points (n = 6). For the current study, data from the baseline questionnaires and the baseline diary study were used. The diary study consisted of 80 items measured once daily for 90 days in the evening by a smartphone questionnaire. Items were chosen based on (a) existing questionnaires and adapted for daily assessment and (b) previous use in diary studies. The specific time in the evening was individualized based on personal preferences and participants had a 90-minute window to complete the diary questionnaire. For more information about the participants, design and procedure, see Booij et al. (2018); for details on the baseline assessments, see Wigman, van der Tuin, van den Berg, Muller, and Booij (2022).

The study was approved by the medical ethical committee of the University Medical Centre Groningen, Groningen, the Netherlands (registration number MEC no. 2015/159, ABR no. NL52974.042.15). The study was conducted in accordance with the Helsinki Declaration. All participants provided written informed consent.

## Measures

### Demographic characterization

For the four subgroups and the total sample, age, gender, and education level are described.

### Baseline questionnaires

#### General psychopathology

The total score of the Dutch symptom checklist revised (SCL-90-R) was used to describe general psychopathology (Arrindell & Ettema, 2003). The SCL-90-R consists of 90 questions concerning different areas of psychopathology and has high reliability (*ω* = 0.98) (Smits, Timmerman, Barelds, & Meijer, 2015) and excellent internal consistency in our sample (Cronbachs Alfa = 0.98).

#### Social functioning

The total score of the Groningse Vragenlijst voor Social Gedrag (Groningse questionnaire for social behavior; GVSG) (Jong & Lubbe, 2001) was used to assess social functioning. This Dutch self-report questionnaires assesses social functioning in nine social domains (parents, partner, having children below and above 15 years, friends, education, paid/unpaid work, household chores, and leisure time).

#### Well-being

The total score of the Flourishing Scale (FS), a short self-report questionnaire, was used to assess psychological well-being (Diener et al., 2010) on several domains. The FS has good reliability and validity (Diener et al., 2010) and good internal consistency in our sample (Cronbachs Alfa = 0.86).

### Diary measures

#### Sleep

Sleep quality and quantity were both measured with one item, every evening about the night before. Sleep quality was assessed with ‘How well did you sleep last night’ scored on a 0–100 Visual Analogue Scale (VAS). Sleep quantity was assessed with ‘How many hours did you sleep last night’ scored in hours and minutes. For the analyses, both the person-mean (pm) and the person-mean centered (pmc) value of sleep quality and quantity were calculated. Pm-sleep was used to examine average differences between individuals in sleep patterns and pmc-sleep to examine within-person effects. Pm-sleep is calculated as the mean value on sleep for 90 days for each individual and pmc-sleep was calculated as the individual's variation around its own pm.

#### Psychotic experiences

Items that fit within the domain of PEs were combined and divided by the number of items (5) to get an average domain score for PEs. The five items were: ‘I felt suspicious today’, ‘Today I had the feeling others disliked me’, ‘I felt that others could read my thoughts today’, ‘I felt unreal today’, and ‘I felt that others could control me today’, and all were scored on a 100-point VAS-scale with a higher score indicating more PEs. Composite reliability of this domain score, which shows whether the items load onto the same scale, was assessed by taking the multilevel structure of the data into account (Geldhof, Preacher, & Zyphur, 2014). The R-package ‘multilevelTools’ (Wiley, 2020) was used to calculate the within- and between-person omega with the function ‘omegaSEM’. The within-person omega was 0.60 and the between-person omega was 0.90. Both the pm- and the pmc-value for PEs was calculated too, identical to the approach for sleep.

### Statistical analyses

All analyses were performed in R version 4.1.3 (R core team, 2022)**.** All multilevel models were created in the R-package ‘nlme’ (Pinheiro, Bates, Debroy, Sarkar, & R Core Team, 2022). An alpha of 0.05 was used as inference criteria.

#### Descriptive analyses

For the four subgroups and the total sample, we calculated descriptive statistics on age, gender, education level, psychopathology, social functioning, well-being, and on daily sleep (quality and quantity) and PEs. As data on sleep and PEs were nested within individuals, the within-person median was first calculated before calculating the median of the subgroups and the total sample. Because of the nested structure, sleep and PEs were compared across the subgroups by means of three multilevel models, one for each domain, with diary domains as the outcomes. We added a centered version of time (day 1–90) as a covariate to control for any trends in the data and a random intercept for individuals.

#### Bidirectional day-to-day relationship between sleep and PEs

To examine the bidirectional day-to-day relationship between sleep and PEs, several multilevel lagged (t-1) models were constructed. For all final models, the method ‘maximum likelihood’ was used as this provides unbiased estimates for the fixed effects. For all tests on the usefulness of random effects, the method ‘restricted maximum likelihood’ was used as this provides unbiased estimates for the random effects. The ‘unstructured’ covariance structure for the random effects was chosen for all models, based on the AIC.

- *Model 1a* assessed the effect of sleep quality from the previous night (measured at *t*) on PEs the following day (measured at *t*). This model had PEs as the outcome, with main fixed effects pmc-sleep quality (*t*) and pm-sleep quality. To control for autocorrelation, PEs (*t-1*) was added as a covariate and to account for any trends in the data, time-centered was added. A random intercept for individuals and random slopes for pmc-sleep quality (*t*), PEs (*t-1*), and time-centered were added to allow for individual variation. The added value of the random slopes for pmc sleep quality (*t*) and PEs (*t-1*) was assessed based on the AIC (with lower values indicating better fit). As both additions improved the model, both were kept.
- *Model 1b* was identical to model 1a with the exception of including sleep quantity as predictor instead of sleep quality. Sleep quantity might have a non-linear relationship with PEs, as more than 8 h sleep might not be beneficial. Therefore, before assessing the relationship between sleep quantity and PEs, we checked whether we saw a clear deviation from a linear relationship between these variables by means of a scatterplot. As there was no clear deviation, we proceeded with sleep quantity without transforming it.
- *Model 1c^1^* combined model 1a and 1b to assess whether effects remained when both sleep quality and sleep quantity from the previous night were predictors of PEs the following day. This model was similar to model 1a, with the addition of pmc-sleep quantity and pm-sleep quantity as fixed effects and pmc-sleep quantity as random slopes.
- *Model 2a* assessed the effect of PEs from the previous day (measured at *t-1*), on sleep quality the following night (measured at *t*). This model had sleep quality as the outcome with main fixed effects pmc PEs (*t-1*) and pm- PEs. To control for autocorrelation, sleep quality (*t-1*) was added as a covariate and to account for any trends in the data, time centered was added. A random intercept for individuals and random slopes for pmc PEs (*t-1*), sleep quality (*t-1*), and time centered were added to allow for individual variation. Both random slopes for pmc PEs (*t-1*) and sleep quality (*t-1*) improved the model and were kept.
- *Model 2b* assessed the effect of PEs from the previous day on sleep quantity the following night. The same multilevel model as model 2a was constructed, except that the random slope for PEs (*t-1*) did not improve the model and thus was not included in the final model.

#### Subgroup differences in the association between sleep and PEs^2^

To assess whether the associations between sleep and PEs differed per subgroup, an interaction term with the pmc-predictor and subgroup (dummy-coded) was added as fixed effect to the models described above. Although we assessed whether adding the interaction term as random effect improved model fit based on the AIC, this did not improve the model and thus were left out. As we compared four groups to each other, we corrected for multiple testing with the False Discovery Rate (FDR) method (Benjamini & Hochberg, 1995).

#### Stratified analyses

In case of a significant interaction effect, the differences between subgroups were assessed with subgroup-specific models of the main effects and the interaction visualized with plots.

#### Power

The power for multilevel models is based on a combination of the number of individuals and the number of observations per individual. For models 1 and 2, we had 96 individuals with 70–90 measurements per individual. Monte Carlo simulations indicated that 96 individuals, a conservative amount of 70 observations per person, a medium effect size (0.3; Cohen, 1988), medium-high ICC of 0.3 (Maas & Hox, 2005) and a medium variance of 0.09 yield a power of 99% to detect an effect for models 1–2. When adding subgroup as an interaction effect to the model, the power to detect an interaction effect was still 0.99. For the stratified analyses, the number of individuals decreased to at least 20 individuals, which reduced the power to 90%. Thus, for all analyses, we expected sufficient power to detect significant effects.

^1^Here we deviate from our preregistration plan. As a post-hoc analysis we assessed whether sleep quality and sleep quantity were still both significant predictors of PEs when combined in one model.^2^ Here we deviate from our preregistration plan. After careful consideration and additional power calculations, we decided to start with a moderation analysis to assess whether subgroups differed significantly from each other, before continuing with stratified analyses.

## Results

### Sample characteristics

The total sample consisted of 96 individuals with a mean age of 24.7 (s.d. = 4.20) years and *N* = 76(%) female. The subgroups did not differ by age, gender or completed education. General psychopathology increased with clinical stage. Social functioning and well-being decreased with clinical stage. Diary measurements showed that within-person mean values of sleep quality and quantity decreased with clinical stage and within-person medians of PEs increased with clinical stage, with especially subgroup 4 (UHR for psychosis) differing from the other subgroups. Participants had on average 8% (range: 0–22%) missing data for all diary items (Table 1).

**Table 1.** Descriptive statistics per subgroup and for the total group

	Subgroup 1 *N* = 25	Subgroup 2 *N* = 27	Subgroup 3 *N* = 24	Subgroup 4 *N* = 20	Total group *N* = 96	Difference^1^
**Cross-sectional measurements**						
*Demographics*						
Age, mean (s.d.)	23.3 (3.38)	24.8 (3.95)	26.1 (4.12)	24.8 (5.28)	24.7 (4.20)	ns
Gender (% Female)	80.0	74.1	70.8	80.0	76.0	ns
Completed education^2^						
Low (%)	4.0	18.5	8.3	30.0	14.5	ns
Middle (%)	56.0	51.9	58.3	50.0	54.2	ns
High (%)	40.0	25.9	29.2	20.0	29.2	ns
Other (%)	0	4.0	4.0	0	2.1	
*Clinical functioning*						
Psychopathology, mean (s.d.)	141.44 (38.24)	173.78 (45.08)	211.04 (56.09)	232.50 (57.29)	186.91 (59.34)	4,3,2 > 1; 4,3 > 2
Functioning, mean (s.d.)	15.33 (1.83)	14.60 (1.71)	13.20 (2.35)	13.00 (1.61)	14.27 (1.89)	3,4 < 1; 3 < 2
Well-being, mean (s.d.)	42.12 (7.75)	35.44 (8.68)	33.33 (8.39)	32.05 (9.05)	35.95 (9.17)	4,3,2 < 1
**Diary items per domain**						
Sleep quality, mean (s.d.)	58.45 (9.30)	50.93 (13.03)	49.72 (10.91)	44.81 (12.33)	51.31 (12.27)	1 > 2, 3,4
Sleep quantity^3^, mean (s.d.)	7.5 (0.51)	7.8 (0.83)	7.1 (1.12)	6.9 (1.59)	7.4 (1.09)	1 > 4; 2 > 3,4
Pes^4^, median (IQR)	6.40 (3.74)	6.56 (6.67)	6.98 (11.27)	22.92 (14.90)	7.83 (9.68)	4 > 1,2,3; 3 > 1

^1^Significant difference *p* < 0.05, ns, not significant; ^2^Low, primary education or lower secondary education; Medium, upper secondary education; High, university/college education; ^3^Measured in hours, ^4^Pes, psychotic experiences.

### Bidirectional day-to-day relationship between sleep and PEs

#### Model 1a: PEs predicted by sleep quality

The random slopes for both pmc sleep quality (*t*) and PEs (*t-1*) improved the model and thus were kept (Table 2). Pmc sleep quality significantly predicted PEs the following day. The negative value indicates that poorer sleep quality preceded more PEs within participants. In addition, PEs on the previous day significantly predicted PEs on the current day, indicating a positive autoregressive effect of PEs. There was no significant effect of general sleep quality on levels of PEs between participants.

**Table 2.** Bidirectional associations between sleep and PEs (model 1 and 2) for the total sample

				95% CI	
Fixed effects	B	Std. error	*p*-value	Lower	Upper
*Model 1a: PEs* (*t*) *predicted by sleep quality* (*t*)					
(intercept)	15.21	3.49			
Sleep quality pmc^1^	−0.03	0.01	<0.001**	−0.04	−0.01
Sleep quality pm^2^	−0.10	0.07	0.15	−0.23	0.03
Pes^3^ (t-1)	0.19	0.02	<0.001**	0.16	0.23
Time centered	0.00	0.01	0.69	−0.01	0.01
*Model 1b: PEs* (*t*) *predicted by sleep quantity* (*t*)					
(intercept)	27.69	5.46			
Sleep quantity pmc	−0.01	0.00	0.001**	−0.01	−0.00
Sleep quantity pm	−0.04	0.01	0.002**	−0.06	−0.02
PEs (t-1)	0.20	0.02	<0.001**	0.16	0.24
Time centered	0.00	0.01	0.77	−0.01	0.01
*Model 1c: PEs* (*t*) *predicted by sleep quality* (*t*) *and sleep quantity* (*t*)					
(intercept)	26.60	5.51			
Sleep quality pmc	−0.02	0.01	0.01*	−0.04	−0.004
Sleep quality pm	−0.01	0.07	0.84	−0.16	0.13
Sleep quantity pmc	−0.00	0.00	0.12	−0.01	0.00
Sleep quantity pm	−0.04	0.01	0.01*	−0.06	−0.01
PEs (t-1)	0.19	0.02	<0.001**	0.16	0.23
Time centered	0.00	0.01	0.75	−0.01	0.02
*Model 2a: Sleep quality* (*t*) *predicted by PEs* (*t-1*)					
(intercept)	48.12	1.82			
PEs pmc (t-1)	0.00	0.03	0.92	−0.06	0.06
PEs pm	−0.20	0.08	0.02*	−0.37	−0.20
Sleep quality (t-1)	0.12	0.02	<0.001**	0.08	0.15
Time centered	−0.00	0.01	0.83	−0.02	0.02
*Model 2b: Sleep quantity* (*t*) *predicted by PEs* (*t-1*)					
(intercept)	438.86	12.83			
PEs pmc (t-1)	0.08	0.14	0.55	−0.19	0.08
PEs pm	−1.06	0.40	0.008**	−1.84	−0.28
Sleep quantity (t-1)	0.05	0.02	0.01*	0.01	0.09
Time centered	−0.02	0.06	0.76	−0.14	0.10

^1^Pmc, person-mean centered; ^2^Pm, person-mean; ^3^Pes, psychotic experiences, *p < 0.05, **p < 0.01.

#### Model 1b: PEs predicted by sleep quantity

Pmc sleep quantity from the previous night significantly predicted PEs at the current day. The negative value indicates that less sleep preceded more PEs within participants. In addition, pm sleep quantity significantly predicted PEs. This effect is negative, meaning that participants who slept less in general, experienced more PEs during the following day. PEs on the previous day significantly predicted PEs on the current day, indicating a positive autoregressive effect of PEs.

#### Model 1c: PEs predicted by sleep quality and quantity

Only Pmc-sleep quality and pm-sleep quantity remained significant in predicting PEs when both sleep quality and sleep quantity were included in the model. Thus, within participants, poorer sleep quality preceded more PEs, and between participants, less sleep predicted of daily levels of PEs.

#### Model 2a: sleep quality predicted by PEs

Within participants, we found no significant effect of PEs on sleep quality. Pm PEs did however significantly predict sleep quality. The negative value indicates that participants who in general reported more PEs experienced poorer sleep quality. Sleep quality on the previous day significantly predicted sleep quality at the current day, indicating a positive autoregressive effect of sleep quality.

#### Model 2b: sleep quantity predicted by PEs

Within participants, we found no significant effect of PEs on sleep quantity. Pm PEs did, however, significantly predict sleep quantity. The negative value indicates that participants who in general experienced more PEs slept less hours on average. Sleep quantity on the previous night significantly predicted sleep quantity at the current day, indicating a positive autoregressive effect of sleep quantity.

### Subgroup differences in the association between sleep and PEs

For all models, we found one significant interaction effect for PEs predicting sleep quality between subgroup 3 and subgroup 4 (*p*_unadjusted_ = 0.02). However, after correcting for multiple testing with the FDR method, this result became non-significant (*p*_adjusted_ = 0.06). Therefore, we did not find robust evidence that the bi-directional association between sleep quality and sleep quantity and PEs differed between different clinical stages on the psychosis continuum. As we did not find any significant interaction effects, we did not proceed with stratified analyses per subgroup.

## Discussion

This study investigated the bi-directional, day-to-day relationship between sleep and PEs over 90 days and assessed whether these associations differed across different early stages along the psychosis continuum, as defined by the clinical staging model (McGorry et al., 2006). We found that both poorer sleep quality and shorter sleep duration the previous night predicted more PEs the following day. When combining both sleep quality and duration in a single model, only poorer sleep quality remained a significant predictor of PEs the following day. Furthermore, based on mean levels of sleep across the diary period, individuals who, in general, slept for shorter periods experienced more PEs on a daily basis. Conversely, we did not find evidence that PEs from the previous day predicted sleep quality or sleep duration the subsequent night within participants. However, looking at patterns over the full 90-day period, we found that individuals who, in general, experienced more PEs, also had poorer sleep quality and shorter sleep duration on a daily basis. We did not find evidence that the relationship between sleep and PEs differed between the four early clinical stages of the psychosis continuum.

Our finding that poorer sleep quality predicted PEs on the following day aligns with several findings from the literature in patients with schizophrenia (Kammerer et al., 2021; Kasanova et al., 2020; Meyer et al., 2021; Mulligan et al., 2016) and individuals from the general population (Hennig & Lincoln, 2018; Hennig et al., 2020). Our study extends these results to individuals in different early stages of the psychosis continuum. We additionally found that shorter sleep duration the previous night was predictive of more PEs the following day. Previous research by Mulligan et al. (2016) reported a relationship between paranoia and sleep quality but not sleep quantity. They therefore concluded that sleep quality was more important than sleep duration in the prediction of next-day paranoia. Consistent with that, we found that, when both sleep quality and quantity were combined into a single model, only sleep quality predicted next day PEs. However, we did find that individuals who in general slept less over a 90-day period experienced more PEs on a daily basis. This indicates that both sleep quality and quantity are important risk factors for the development of PEs, albeit on a different scale. For sleep quality, fluctuations at a daily level seem more strongly associated with PEs and for sleep quantity, sleep patterns over a longer period of time seem more strongly associated with PEs. Importantly, our study also adds to the previous literature by showing that the relationships between PEs and sleep quality and quantity can be detected over a longer period of time (90 days) and that the relationship is not merely a result of autocorrelation of PEs (i.e. PEs from the previous day predicting PEs on the following day). An important additional benefit of such a longer period is that the assessment of sleep is more representative of one's overall sleeping pattern and less prone to occasional sleep changes.

We found no evidence that PEs from the previous day predicted sleep quality or duration the following night. This is in contrast with a study by Meyer et al. (2021) who found that PEs did predict sleep quality in individuals with schizophrenia, albeit weaker than the effect of sleep on subsequent PEs. In addition, Hennig et al. (2020) found that paranoid symptoms predicted less restful sleep and more negative dreams in a general population sample. In line with our results, Hennig and Lincoln (2018) and Kasanova et al. (2020) found that PEs did not predict sleep quality or duration the following night. While we did not find that daily fluctuations in PEs predicted daily sleep quality or quantity, we did find that people who in general experienced more PEs, generally had poorer sleep quality and shorter sleep durations on a daily basis. This implies that it is not so much within-person fluctuations in PEs that influence sleep quality and duration, but rather high stable levels of PEs over time. One possibility is that the effect of PEs on sleep is based on a built up of PEs symptoms that only result in poorer sleep when persisting for a longer period. This would underline the importance of decreasing PEs to improve sleep quality and quantity, which, in turn, promotes further decreased PEs (as shown by sleep quality and quantity predicting PEs on a daily basis). However, it is also possible that, due to the relatively low level of PEs in our subclinical sample, fluctuations in PEs were too small to lead to poorer sleep. The exact mechanism driving the relationship between sleep problems and PE is unclear (Waite et al., 2020); however, some potential explanations have been proposed. Negative affect has been found to be a consistent mediator between sleep problems and PEs (e.g. Freeman et al., 2010; Freeman et al., 2017; Kasanova et al. 2020; Reeve et al. 2018*b*). Another mechanism that has been proposed runs through a developmental diathesis-stress model where sleep dysfunction can lead to both biological and psychological stress responses which can trigger the development of PEs (Lunsford-Avery & Mittal, 2013; Lunsford-Avery et al., 2015; Wang et al., 2023). In conclusion, while we did not find evidence for a day-to-day downwards spiral between poor sleep and PEs, both reinforcing each other on a daily basis, we did find that higher average levels of PEs predicted poorer sleep, and in turn, poorer sleep predicted more PEs on a daily basis.

We did not find differences in the relationship between sleep and PEs across early clinical stages of the psychosis continuum. This implies that, even though the mean scores of both PEs and sleep differed across the stages, the associations between the two were comparable across stages. This suggests that sleep problems may be a risk marker in the development of PEs in the early clinical stages of psychosis. However, an alternative explanation for our finding is that our subgroups along the early stages of the psychosis continuum differed from each other in several ways that may have influenced the day-to-day association between sleep and PEs. Individuals from subgroups 2–4 attended mental health services, in contrast to individuals from subgroup 1, and were exposed to a variety of treatments. The variation in treatment was too large, and the level of detail on specific treatments too low, to take this into account in the analyses.

Our results should be viewed in light of some strengths and limitations. First, our data allowed us to look at temporal relationships because sleep and PEs were measured daily. However, this does not necessarily prove causation. Nonetheless, our findings show, at the least, that poorer sleep may be a valuable risk marker, if not a casual factor, in the development of PEs. This is in line with a study by Freeman et al. (2017) that showed that treating insomnia in a large sample of college students decreased paranoia and hallucinatory experiences. Another consideration is that sleep was assessed retrospectively as sleep from the previous night was assessed during the following evening and with self-report, increasing the risk of recall bias. To minimize patient burden, questions were asked only once a day, at the end of the day. Research has shown that subjective and objective measures of sleep align well in adolescents (Lucas-Thompson, Crain, & Brossoit, 2021), and more specifically in patients with schizophrenia (Mulligan et al., 2016). Nevertheless, combining subjective sleep measures with objective measures like actigraphy could provide a more complete picture. Several other factors could have confounded the associations between sleep and PEs as well as differences in the associations between subgroups. These include medication use, substance use and other psychiatric disorders. Medication use can impact sleep both positively (when treating insomnia) and negatively (as an unwanted side-effect). As individuals in subgroup 4 were UHR for psychosis, they were more likely to receive anti-psychotic medication. This may have potentially masked an altered sleep – PE associations in this subgroup. As this cannot be excluded, generalization of the findings should be considered carefully. In addition, we did not control for life events during the diary period, which could have influenced our results. Due to our long measurement period of 90 days, however, we expect that minor life events should not have had a large overall effect on our results. It is important to keep in mind that while we used the general term ‘psychotic experiences’, this did not encompass all forms of PEs but focused mainly on paranoid ideas and bizarre experiences. While we did have information about hallucinations, we excluded them as they were very rarely endorsed, making the data highly skewed and adding little to no information to the analyses. In addition, including the hallucination items drastically decreased the composite reliability of the ‘psychotic experiences’ domain indicating that they did not belong to the same scale.

## Conclusion

In a longitudinal study of people at different early stages of the psychosis continuum, we found that, within individuals, poorer sleep quality predicted next day PEs. We did not find, conversely, that PEs predicted poorer sleep quality or shorter sleep duration the subsequent night. We additionally found that individuals who had generally shorter sleep duration patterns experienced more PEs on a daily basis and that individuals who had generally more PEs had poorer sleep quality and shorter sleep durations on a daily basis. Overall, our results highlight the importance of assessing sleep as an important risk marker in the early clinical stages for psychosis. This implies that clinicians should be extra alert when patient along the early clinical stages present sleep problems as these are related to an increase in PEs. Previous studies have shown that treating insomnia with cognitive behavioral therapy is effective in reducing PEs in individuals with schizophrenia (Freeman et al., 2015), individuals at UHR for psychosis (Bradley et al., 2018) and the general population (Freeman et al., 2017). Our results underline the importance of future intervention research to assess whether improving sleep quality or duration might also reduce PEs in individuals in the early clinical stages, before stage 1b (UHR).
